# Response: Commentary: A construct divided: prosocial behavior as helping, sharing, and comforting subtypes

**DOI:** 10.3389/fpsyg.2018.00553

**Published:** 2018-04-17

**Authors:** Jonathan S. Beier, Kristen A. Dunfield

**Affiliations:** ^1^Department of Psychology, University of Maryland, College Park, MD, United States; ^2^Department of Psychology, Concordia University, Montreal, QC, Canada

**Keywords:** prosocial behavior, socio-cognitive development, emotional development, commentary, early childhood

Tunçgenç (2016) raises important considerations regarding Dunfield's (2014) framework for understanding early prosocial development, offering critical insights about the framework's ecological validity and the role of emotional distress cues. Here, we show how these observations both clarify the scope of Dunfield's framework and underscore its value as a theoretical tool.

According to Dunfield, to act prosocially, children must represent and respond to the specific type of negative state another agent is experiencing. She proposes that there are three varieties of negatives states (i.e., instrumental needs, material desires, and emotional distress), which are addressed by utilizing dissociable sets of cognitive abilities (e.g., representing goals, material desires, and emotions, respectively). Yet, as Tunçgenç observes, many real-world problems involve the co-occurrence of multiple negative states. As anyone who has lost their wallet knows, a failed search is often followed closely by material desire and emotional distress. Because these negative states derive from different aspects of the same event, there is an important question of how to respond. Should one help in the search, share some funds, or comfort the distressed owner?

Tunçgenç asserts that, because the negative states are intermeshed, there are a variety of “right” responses. Although we agree that real-world scenarios often involve the presentation of multiple negative states, we do not agree that all solutions are equally good. When prosocial actors encounter an individual experiencing multiple negative states, we suggest that they will seek to maximize the relief they provide. Thus, the “right” response is a psychological judgment made by a specific prosocial actor, shaped by that actor's values, beliefs, and abilities to generate different possible responses and evaluate their likely efficacies.

A closer look at Tunçgenç's wallet example reveals how the social-cognitive abilities described in Dunfield's framework can inform children's evaluations of possible responses. One consideration is whether there is an underlying problem whose solution would resolve multiple negative states. Giving money (sharing) or offering sympathy (comforting) might provide the recipient some immediate relief. However, the owner may still experience additional hurt as she recalls items to be replaced, or require additional donations when the shared funds run out. A child who appreciates the dependencies between different negative states may conclude that helping to find the lost wallet should be the top priority. Alternatively, children who believe that continued search is unlikely to succeed may shift their response from helping to comforting. Another possibility is that children will offer verbal reassurance while assisting the search. More generally, we expect that children's developing prosocial responses will draw on increasingly sophisticated decision rules for determining what the most effective or appropriate aid would be, given the urgency of current and anticipated needs, within the constraints of what is possible to provide.

To our knowledge, no empirical research has examined how or when children begin to triage others' multiple needs—but there are hints in the literature that they have some requisite abilities. Children readily distinguish between a person's proximate and ultimate goals (e.g., by handing over a functional mug instead of the broken one to which a person is reaching), indicating they are attuned to the root of a problem (Martin and Olson, 2013). Children also identify those most capable of responding to an instrumental need (e.g., tall people are better for reaching than short people), indicating they consider the efficacy of one's available means of helping (Paulus and Moore, 2011). Yet no studies have investigated whether children, or adults, consider the perceived efficacy of different responses to multiple ongoing negative states. Under what circumstances do children choose to help vs. comfort? Do they weigh the benefit of short-term relief against a more long-term solution? To what extent do individuals incorporate their likelihood of success into decisions about how to respond? And do they consider a recipient's preferences for particular solutions? These are exciting directions for new research.

Tunçgenç's second point focuses on the role of emotional distress in the production of children's prosocial behavior. She suggests that, “most empirical studies have incorporated *emotional distress* while testing helping and/or sharing behavior” (Tunçgenç, 2016), concluding that it is unclear to what extent children's responses to a problem result from recognizing emotional distress versus an instrumental need or a material desire. We agree that distress can influence children's prosocial responding, but not as Tunçgenç describes. Emotional cues can signal that a problem has occurred: If a child is not paying close attention, or is unfamiliar with what a person is trying to do, emotional expressions such as frustration can capture attention and highlight that a problem is happening (e.g., Brownell et al., 2009). Emotional cues also communicate the intensity of a negative state (e.g., Svetlova et al., 2010), which may support children's decisions about when and how to respond. However, emotional cues alone do not reveal what the problem is or how to solve it—especially if the problem is rooted in the instrumental or material domain. In this critical sense, expressions of frustration from a failed goal or of sadness from material desire are an *insufficient* basis for effective prosocial responding. A major contribution of Dunfield's framework is its inventory of the cognitive skills required for understanding the causal structure of the problem that produced those unpleasant feelings: Why does the person feel that way and what is an effective means to address it?

Although emotional distress can be a downstream consequence of instrumental need and material desire, several lines of research argue against the *necessity* of emotional cues in eliciting children's helping or sharing. First, infants represent and care about the goals of non-human, geometrically-shaped agents (Hamlin, 2015), and, as Tunçgenç observes, they even help these unfamiliar agents when their goals are unfulfilled (Kenward and Gredebäck, 2013). Second, children help proactively in anticipation of a person's predicted goal (Warneken, 2013). In both cases, children's prosocial motivations do not depend upon a frustrated agent's emotional displays. Finally, adding emotional distress to an instrumental need does not increase helping (Newton et al., 2014). Because emotional distress is neither sufficient nor necessary for children to initiate helping (and likely sharing) responses, its presence in some experimental tasks does not obviate the requirement that participants recognize an individual's instrumental need (or material desire).

By responding to Tunçgenç's (2016) thoughtful observations, we hope to have highlighted the gap between the original aims of Dunfield's framework (2014)—to delineate the problem spaces of different types of prosocial behavior and to identify the cognitive skills required to address each one—and its application to real-world scenarios. Naturalistic prosocial behavior is clearly more complicated than structured observations often presume (Dirks et al., 2018), and the potential for multiple ongoing negative states and the supplemental information that emotional distress can offer are important topics for future research to examine. We suggest that this will be most fruitful as part of a larger research program investigating decision-making processes underlying children's choices about when and how to act prosocially, a program that will continue to benefit from the framework offered by Dunfield.

## Author contributions

Both authors made equal intellectual contributions to this piece and approved it for publication.

### Conflict of interest statement

The authors declare that the research was conducted in the absence of any commercial or financial relationships that could be construed as a potential conflict of interest.
